# Endocrine regulation and metabolic mechanisms of osteopontin in the development and progression of osteosarcoma, metastasis and prognosis

**DOI:** 10.3389/fendo.2022.1100063

**Published:** 2023-01-13

**Authors:** Zhuce Shao, Shuxiong Bi

**Affiliations:** Third Hospital of Shanxi Medical University, Shanxi Bethune Hospital, Shanxi Academy of Medical Sciences, Tongji Shanxi Hospital, Taiyuan, China

**Keywords:** osteopontin, osteosarcoma, endocrine regulation, metabolism, cancer

## Abstract

Osteosarcoma is the most common type of malignant bone tumor, occurring in adolescents and patients over 60. It has a bimodal onset and a poor prognosis, and its development has not yet been fully explained. Osteopontin (OPN) is a high protein consisting of 314 amino acid residues with a negative charge and is involved in many biological activities. OPN is not only an essential part of the regulation of the nervous system and endocrine metabolism of skeletal cells. Still, it is also involved in several other important biological activities, such as the division, transformation, and proliferation of skeletal cells and their associated cells, such as bone tumor cells, including bone marrow mesenchymal stem cells, hematopoietic stem cells, osteoblasts, and osteoclasts. Osteoblasts and osteocytes. Recent studies have shown a strong correlation between OPN and the development and progression of many skeletal diseases, such as osteosarcoma and rheumatoid arthritis. This review aims to understand the mechanisms and advances in the role of OPN as a factor in the development, progression, metastasis, and prognosis of osteosarcoma in an attempt to provide a comprehensive summary of the mechanisms by which OPN regulates osteosarcoma progression and in the hope of contributing to the advancement of osteosarcoma research and clinical treatment.

## Introduction

Osteosarcoma (OS) is the most common primary skeletal-related malignancy in young people. It is the second leading cause of cancer death in children and adolescents (1),always presenting as growth in tubular long bones and giving rise to less differentiated skeletal cells (2, 3). Osteosarcoma is characterized by a bimodal pattern, with the first peak occurring in children and adolescents, and the second peak occurring in patients over 60 years of age (4). About half of these patients have tumors near the knee (5), and osteosarcoma has a high metastasis rate of nearly 20%, with the lungs and lymph nodes being the frequent sites of metastasis (6), and metastasis has a severe impact on the patient’s prognosis.

Osteosarcoma cells share many similarities with primitive bone cells, such as a strong proliferative capacity and resistance to apoptosis. Also, they produce components such as connective tissue growth factor, runt-related transcription factor 2 (RUNX2), alkaline phosphatase (ALP), and osteocalcin. Osteopontin is overexpressed in many tumors and may have a strong correlation with the development, metastasis, and prognosis of many types of tumors. For example, one study found that osteopontin in lung cancer, a rise in osteopontin was associated with the survival and prognosis of lung cancer patients (7–16). Osteopontin predicts poor prognostic performance after neoadjuvant chemotherapy for breast cancer (17–22). In addition, the upregulation of osteopontin concentrations *in vivo* was associated with tumor metastasis in gastrointestinal cancer, and even with the size and grade of the tumor (23–41). Significant correlation between osteopontin and drug resistance in urological tumors (42–54). Of course, osteopontin is also closely related to osteosarcoma, and we will systematically review and describe osteopontin and its endocrine and metabolic mechanisms in relation to osteosarcoma.

To describe the structural function of OPN and the possible mechanisms by which OPN regulates the development, progression, metastasis, and prognosis of osteosarcoma

**Figure 1:**
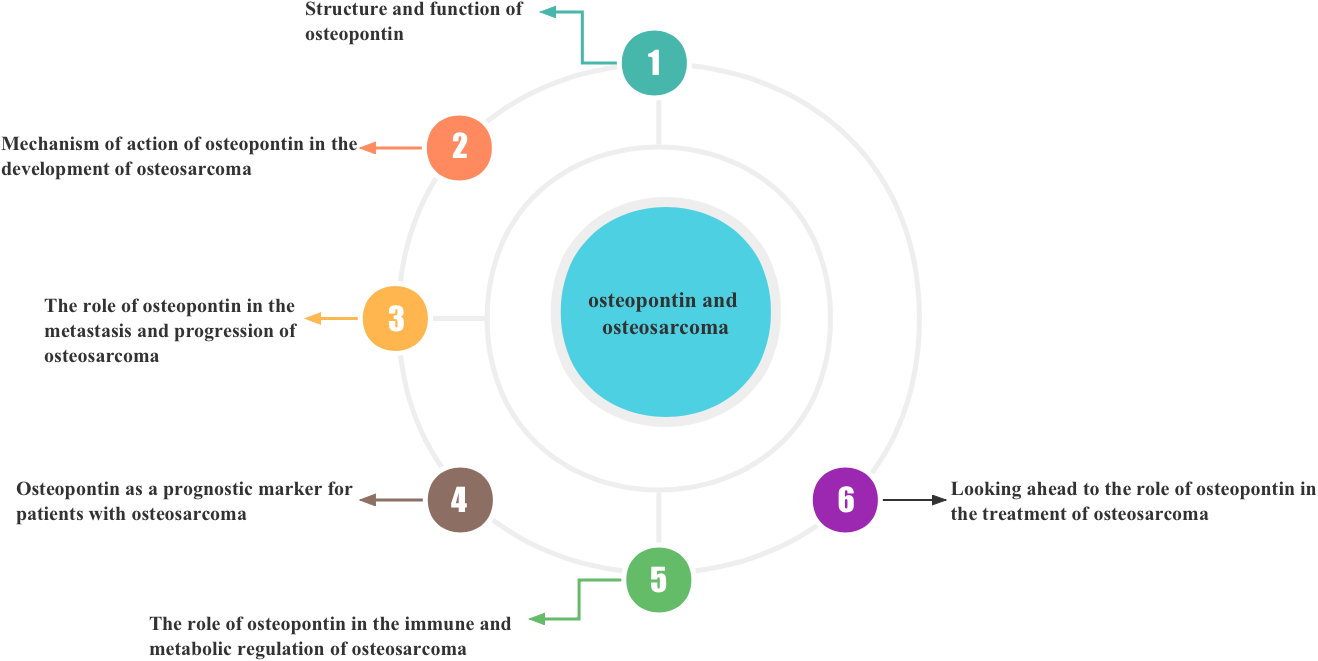
Guide map.

In the following sections, we will discuss the structure and function of osteopontin and, in addition, provide as comprehensive an understanding as possible of the possible role of osteopontin in the development, progression, metastasis, and prognosis of osteosarcoma. An attempt is made to summarise the mechanisms by which OPN regulates osteosarcoma and can contribute to the progress of osteosarcoma research and clinical treatment. In addition, **Figure 1** details the sequence and content of what we will describe next.

## Function and structure of osteopontin

**Figure 2:**
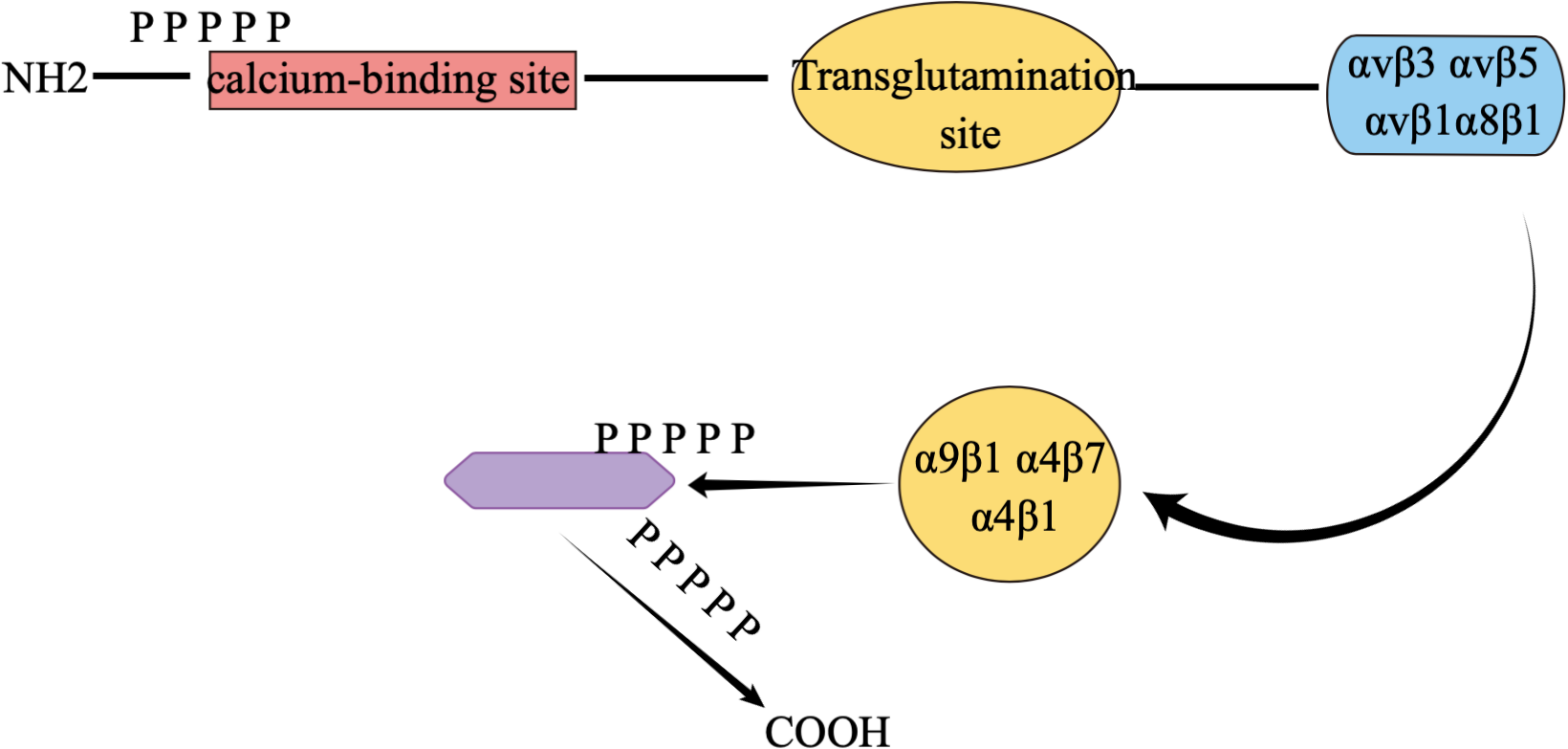
Concise molecular formula of osteopontin. αvβ1, αvβ3, αvβ5, α8β1, α9β1, α4β7 and α4β1 are all different integrins of osteopontin, which are responsible for interacting with cells.

**Figure 3:**
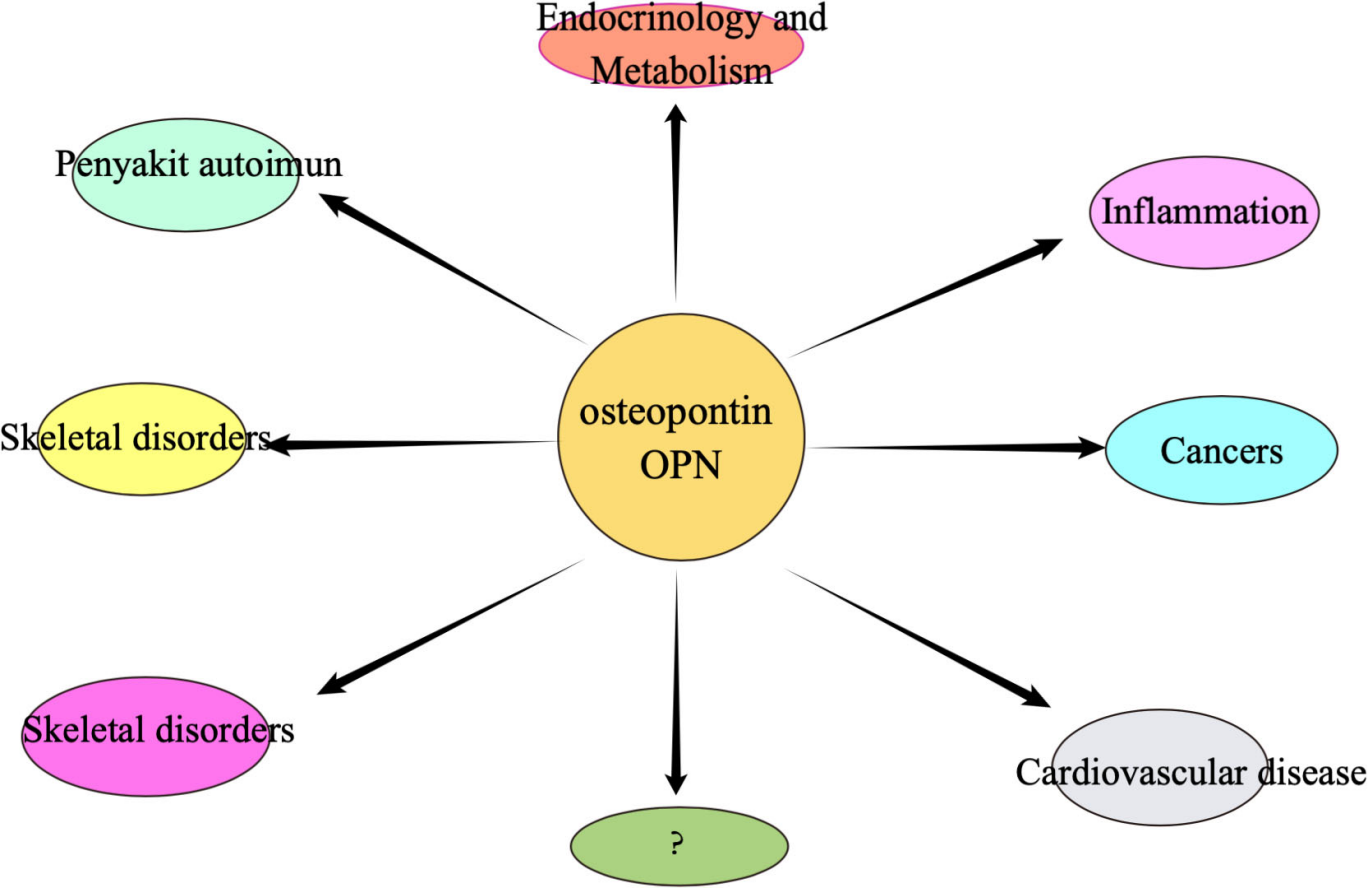
Osteopontin may be associated with many of the different types of diseases mentioned above.

Osteopontin is an extracellular matrix (ECM)-associated, rich phosphoglycoprotein (55–57). Osteopontin (OPN) is a negatively charged glycophosphoprotein with a high content of aspartic acid, composed of 314 amino acids and with acidic properties (58, 59), which was first identified in bone. There are five isoforms of OPN, and, to our knowledge, high expression of OPN is found in various tissues such as skin, kidney, bone, and teeth, as well as in some cancer cells, including blood. Numerous studies have identified a crucial role of OPN in early life development (60–65). The molecular structure of osteopontin is rough as shown in **Figure 2**. osteopontin is implicated in various diseases or mechanisms of action, which we have briefly described in **Figure 3**.

## Effect of osteopontin on the initial onset of osteosarcoma

Many studies in recent years have demonstrated a clear correlation between osteopontin and the initial development of osteosarcoma. Downregulation of osteopontin levels can prevent mesenchymal stem cells or immature osteoblasts from progressing to mature cells, allowing them to maintain the morphology and characteristics of immature primitive cells, which may ultimately lead to the development of osteosarcoma (66–69).

In addition, the glucose transporter is one of the regulators of osteosarcoma growth (70, 71), upregulating levels through the hypoxia-induced pathway and thereby adapting to hypoxia and increasing tissue oxygenation (72–75).

**Figure 4:**
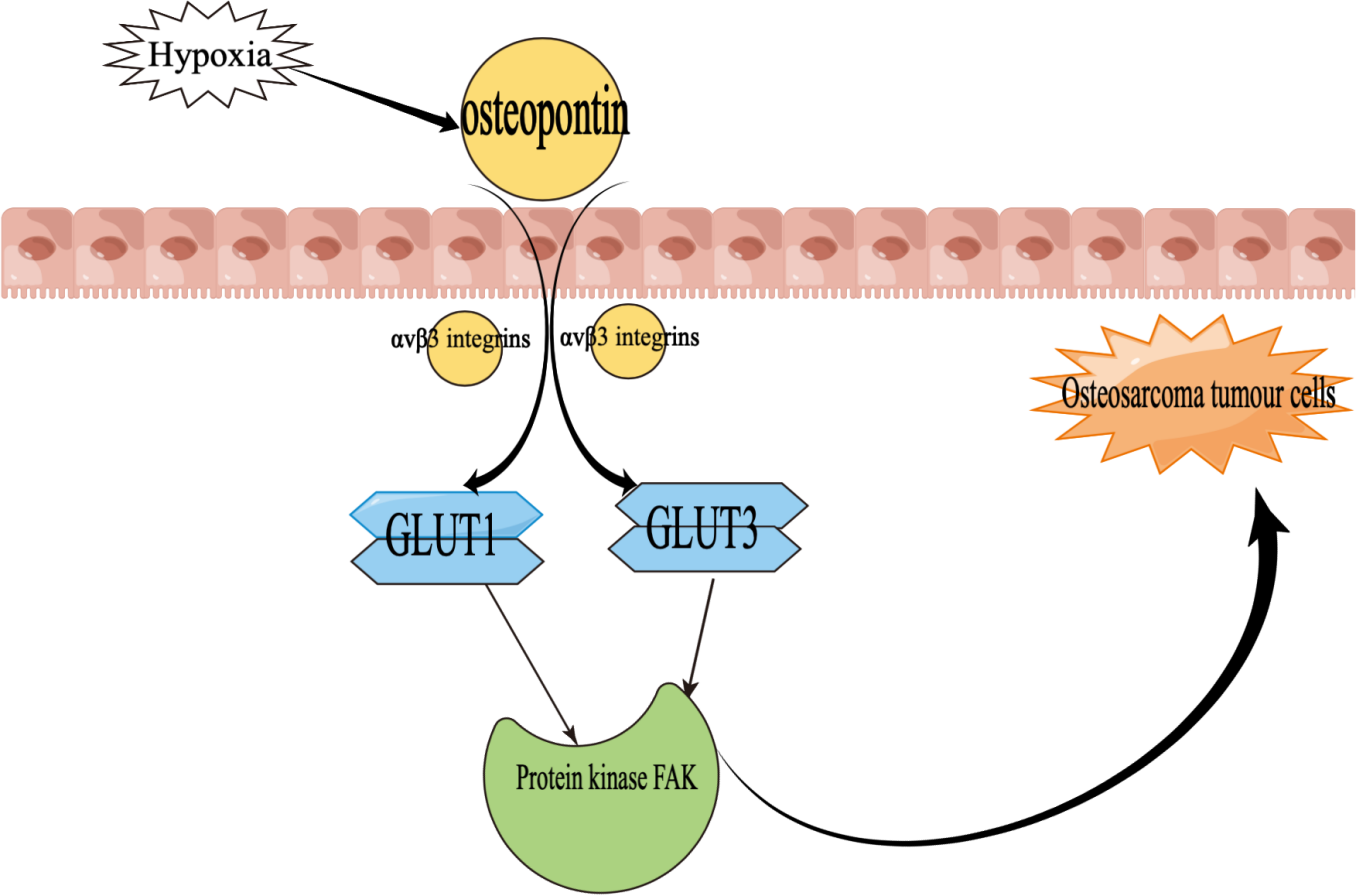
Mechanisms of the role of osteopontin in osteosarcoma development GLUT1, glucose transporter 1; GLUT3, glucose transporter 3. Focal Adhesion Kinase (FAK) is a tyrosine kinase that plays an important role in the evolution of osteosarcoma.

Hypoxia induces upregulation of osteopontin, which then increases GLUT1 and GLUT3 protein expression mediated by αvβ3 integrins and ultimately activates the protein kinase FAK pathway, leading to the initial development of osteosarcoma step by step (76). In particular, **Figure 4** demonstrates the mechanism of action of osteopontin in the development of osteosarcoma.

## The role and impact of osteopontin in the development of metastasis in osteosarcoma

**Figure 5:**
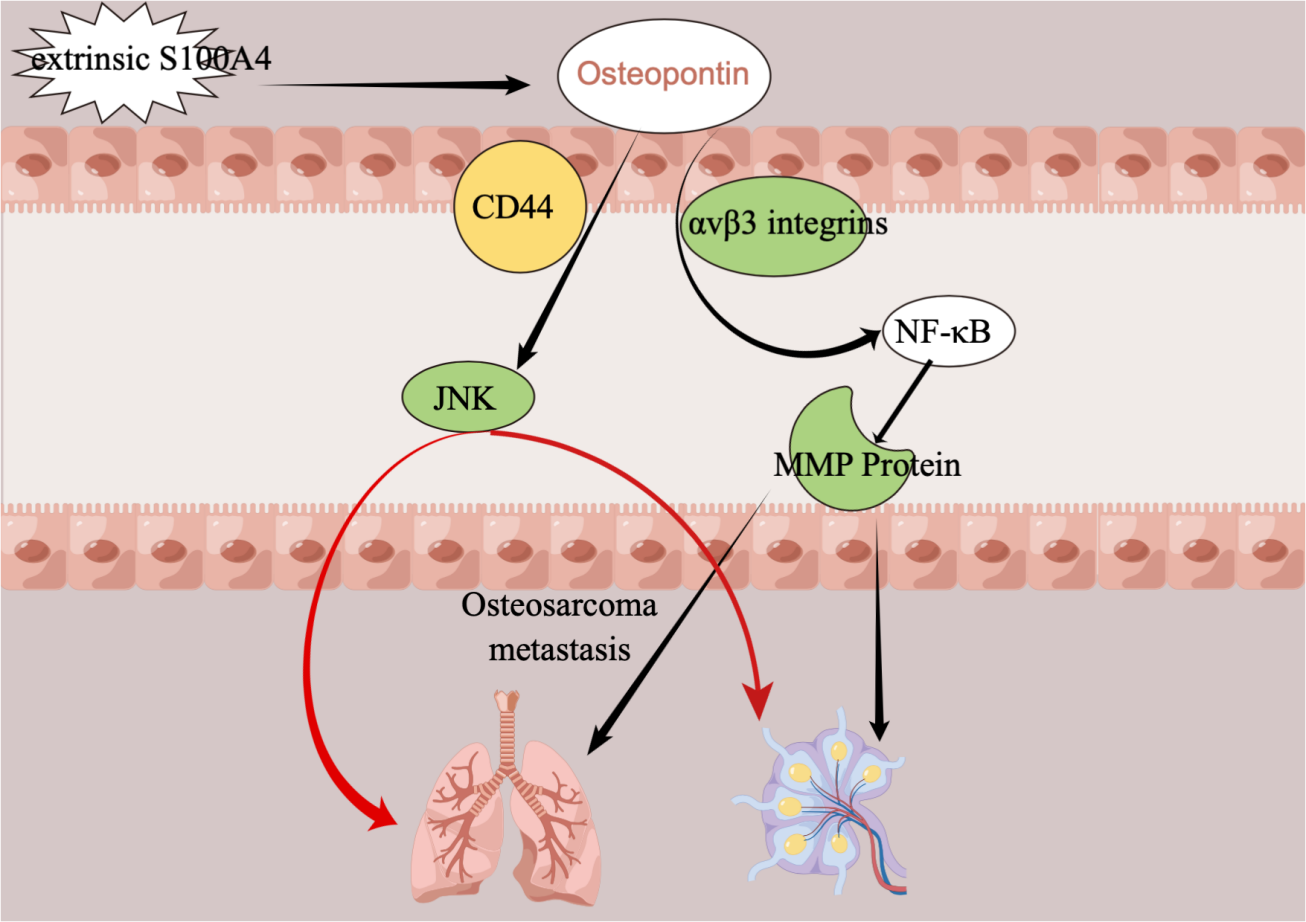
The mechanism of action of OPN in the metastatic process of osteosarcoma. CD44, extracellular matrix receptor III; MMP, matrix metalloproteinase; NF-κB, nuclear factor-κB; JNK, c-JUN N-terminal kinase.

Metastases from osteosarcoma, most commonly in the lungs, are a key determinant of the lethality of osteosarcoma (77–79). It is, therefore, particularly important that we find ways to understand some or even all of the channels or mechanisms of osteosarcoma metastasis so that we can find ways to stop or disrupt the processes and pathways of osteosarcoma metastasis. The mechanism of metastasis and the various factors influencing it have not been well studied, but the role of OPN in the process of tumor metastasis in osteosarcoma can be elaborated and explained graphically. Several studies have demonstrated that S100A4 protein is associated with the metastasis of cancers, including osteosarcoma, that it increases the tumor metastatic capacity of cancers (80–83), and that S100A4 protein can even be a potential marker for predicting cancer metastasis (84, 85). And the effect of the S100A4 protein on tumor metastasis in osteosarcoma is also accomplished through a transition in regulating OPN levels. So, how exactly do S100A4 protein and OPN link and affect osteosarcoma metastasis? The study found that the extracellular S100A4 protein has been shown to activate NF-κB (86). Osteopontin was previously found to have elements that respond to NF-κB (87), so it was later shown that initially, the S100A4 protein regulated osteopontin horizontally, then linked to NF-κB through osteopontin, and finally led to the horizontal regulation of MMP protein (88, 89). This series of changes may eventually lead to the development of metastases in osteosarcoma, with the most likely organs of osteosarcoma being the lungs and lymphatic tissues. The role of Runt-related transcription factor 2 (Runx2) is also important in the metastatic process of many cancers, including osteosarcoma (90–95). Recent studies have shown that Runx2 in combination with osteopontin promotes the adhesion of osteosarcoma cells to the cell surface of the lung, an important step in the distant metastasis of osteosarcoma to the lung (96). In addition, **Figure 5** demonstrates the mechanism of action of osteopontin in the metastatic process of osteosarcoma.

## Osteopontin as a prognostic marker for patients with osteosarcoma

Osteosarcoma is a highly malignant tumor, and many patients develop early metastases and have a terrible prognosis. Not only is OPN a marker for the development and metastasis of many different tumors, but even changes in OPN levels can correlate strongly with the prognosis of patients with osteosarcoma (97). In the study by Wong IH et al., mRNA levels of OPN were increased in more than 90% of patients with osteosarcoma, in addition to the healthy population but only a tiny proportion. Their study also suggests that peripheral blood OPN levels can be used as a predictive assessment for patients with osteosarcoma (98). In addition, one study differed from the above results. Firstly, OPN and vascular endothelial growth factor (VEGF) constitute a vascular protein. In their study, the expression of OPN in benign and malignant bone tumors was determined, and the prognostic effect of OPN expression on the outcome of osteosarcoma patients was studied. Express OPN and VEGF, the final results showed that OPN expression had no effect on patients’ overall or disease-free survival. Although the expression of OPN is associated with the expression of VEGF in osteosarcoma, the change of OPN level does not predict the good or bad prognosis of osteosarcoma patients (99).

## The role of osteopontin in the immune and metabolic regulation of osteosarcoma

The intracellular osteopontin mRNA synthesizes two types of osteopontin, secreted osteopontin (sOPN) and intracellular osteopontin (iOPN). During immunotherapy of cancer, including osteosarcoma, tumor cells sometimes produce an immune escape, with the end result that cancer cells remain, and consequently, tumor metastasis occurs. The immunomodulatory effects of osteopontin include the development of osteosarcoma and distant metastasis and further cause the development of immunosuppression at the site of metastasis (100). It has been found that interferon regulatory factor 8 associates with osteopontin and causes a downregulation of osteopontin levels, which then activates T cells, meaning that interferon regulatory factor 8 levels are negatively correlated with osteopontin levels and that a decrease in interferon regulatory factor 8 levels leads to an upregulation of osteopontin levels, which can then reduce or even block the activation of T cells, which ultimately leads to immune escape from cancer, including osteosarcoma (101). Some studies have been conducted to discover the mechanism of action of osteopontin in the immunometabolism of certain cancers and their metastasis (102, 103). Detailed information on the mechanisms of endocrine and metabolic action of osteopontin in the development, progression, and metastasis of osteosarcoma and its regulation at the cellular level remains to be explored.

## Looking ahead to a more valuable role for osteopontin in the treatment of osteosarcoma

OPN is involved in bone development and metabolism in the development and expansion of skeletal diseases through endocrinology and immunity. At the cellular level, OPN is involved in more refined activities through signaling pathways. Although studies in recent years have also produced many results on the association of OPN with the occurrence, development, metastasis, and prognosis of osteosarcoma, many of the mechanisms are still obscure. The in-depth study of OPN provides new ideas and directions for the interpretation of the pathogenesis of osteosarcoma. It gives a new target for the treating crucial clinical significance and value. We hope that future studies can better understand the mechanism of OPN’s role in the occurrence and development of osteosarcoma, including improving clinical prognosis, etc. We wish to interfere with or intervene in advance of the function of OPN in osteosarcoma and slow or even block the progression of osteosarcoma in the future to alleviate the pain of osteosarcoma patients and improve their disappointing survival rate.

## Discussion

Cancer has become a global problem that cannot be ignored— it is one of the most common causes of death among older adults, with a high mortality rate from osteosarcoma. Over 3600 new bone cancer diagnoses and 1720 deaths from bone cancer occur every year in the United States (104). Distant metastases from osteosarcoma occur as a result of hematogenous spread, with the most significant probability occurring firstly in the lungs and secondly in the lymphatic system. These metastases are strongly associated with a poor prognosis. Osteosarcoma cells show a high propensity to spread and a relatively high likelihood of distant metastases. Osteosarcoma can metastasize to almost any organ, so the prognosis for patients with osteosarcoma is always dismal. Metastatic osteosarcoma cells settle and grow in a second organ later on and eventually develop into a metastatic lesion. The cell cycle undergoes differentiation, metabolism, and the formation of a new microenvironment suitable for the growth of metastatic osteosarcoma cells, and also, the metastatic cells are not identical to the original osteosarcoma cells (105, 106). There has also been notable success in the extensive research over the years to discover the underlying mechanisms by which osteosarcoma develops distant metastases. The tireless efforts of medical scientists have led to the discovery of additional markers involved in osteosarcoma metastasis-related metastases, followed by numerous cellular or animal studies that have further validated a number of relevant genes and pathways (107–110). Later, based on the results of these basic experiments, many clinical studies were subsequently conducted to improve treatment modalities. There is a wide variety of genes and proteins involved in the pathways and mechanisms by which osteosarcoma develops distant metastases, and a wide variety of genes and proteins involved in osteosarcoma metastasis, in which OPN must play an important role, We also address in this review only the role of OPN in the development and metastasis of bone tumors.

It has been found that osteopontin regulates cell signaling by binding to receptors that ultimately affect or directly contribute to tumor cell growth and metastasis, of which the main osteopontin receptors include integrins and CD44 (111). The broad metabolic pathways of action of osteopontin are also described in the section of the manuscript above. With the ongoing results of research on osteopontin, it is promising that we seem to be seeing alternative avenues for the pathogenesis and treatment of osteosarcoma. For example, reducing the expression of OPN levels may provide new strategies for the treatment of various types of metastatic cancers (112). In recent studies, Zhang et al. (66) demonstrated that hyperoside regulates OPN by inducing a cell cycle arrest and can hinder the development of osteosarcoma cells and promote further differentiation of osteosarcoma cells into osteoblasts. In addition, it has been suggested that VD3 upregulates OPN by activating cell cycle inhibitors such as p21 (113), thereby promoting osteopontin differentiation into osteoblasts. These may be new strategies for the treatment of OS in the future (114).

## Conclusions

OPN can be secreted by many tissues and has many controversial effects on health. It can be argued that there is no clear answer to the metabolic and immunological effects of OPN on inflammation or cancer. In addition, the treatment of osteosarcoma, the most common primary bone malignancy, has been a challenge, and modern developments in molecular medicine have led to the discovery of several potential tumor markers. Recent studies have sought to use OPN as a diagnostic and prognostic marker for osteosarcoma to monitor the developmental status of osteosarcoma and assess its therapeutic efficacy. To better understand the role of OPN in the development and metastasis of osteosarcoma and to provide the basis for new therapeutic approaches to treat this life-threatening disease, more evidence from cellular studies and subsequent clinical trials is needed and will be awaited.

## Author contributions

ZS designed and conceived the study and wrote this paper. SB revised the article. All authors contributed to the article and approved the submitted version.
